# Who Is Afraid of Population Aging? Myths, Challenges and an Open Question from the Civil Economy Perspective

**DOI:** 10.3390/ijerph17155277

**Published:** 2020-07-22

**Authors:** Iñigo Calvo-Sotomayor, Ekhi Atutxa, Ricardo Aguado

**Affiliations:** 1 Department of Strategy and Information Systems, University of Deusto, 48007 Bilbao, Spain; 2 Department of Economics, University of Deusto, 48007 Bilbao, Spain; ekhi.atutxa@deusto.es (E.A.); ricardo.aguado@deusto.es (R.A.)

**Keywords:** aging, myths, civil economy, productivity

## Abstract

Population aging is a great human achievement, but the economic literature normally addresses its effects in a narrow way and as a “problem” to be solved. The objective of this paper is to provide a more balanced approach to aging by calling into question some widespread ideas in the economic literature on aging, such as its supposed negative influence on economic growth, its impact on labor productivity or the assumption that aging societies are incapable of applying reforms. The paper adopts the renewed civil economy framework and takes as a reference the existing literature about beliefs and wrong assumptions on aging. The innovative contribution of this analysis lies in its effort to foster a positive perspective in the population aging field of research and in challenging negative associations regarding old-age stereotypes.

## 1. Introduction

Population aging is one of the greatest achievements in recent human history. A phenomenon produced by the combination of low fertility rates with sustained increases in average life expectancy. A demographic success history that can be seen as a consequence of economic development, social welfare and the advancement on health and medical technologies [1,2,3].

Even if population aging is the product of great social, political and economic advances, it is common for economic research to look at it as a problem to be solved. Negative references in the literature on demographic aging are well documented, so much that it is not unusual to find terms as "demographic burden" [4] (p. 429), [5] to explain the negative economic consequences of an aging population, or “demographic time bomb” [6,7], to describe the progressive increase in the average age of the workforce. More broadly and beyond the economic perspective, expressions such as "demographic suicide" appear in the literature [8] regarding the declining fertility rate in Europe, or "demographic winter", a term used by pro-natalist movements [9]. Some authors even predicted the advent of an "age war" in the future [10].

From a historical perspective, this way of describing, understanding and explaining demographic changes and their consequences has led to what Kelley [11] calls an "alarmist" conception of demographic analysis. A conception followed by researchers who claimed, for example, that the rapid population growth experienced during the 20th century was going to bring a series of disastrous consequences. Schindlmayr [12] (p. 37) called it the “population hysteria” of the 1960s and 1970s. However, these predictions never materialized [13], although the “demographic explosion” has been affecting various economic, social and political aspects. On the contrary, the National Research Council [14] established a non-alarmist or "revisionist" position when carrying out population growth research. It may be interesting to consider this position in the study of aging, given that not only this field of knowledge is full of “myths” [15,16] (p. 407) but also because negative associations still prevail regarding old-age stereotypes [17].

It is evident that population aging causes a wide range of socio-economic effects when it appears [18,19], since the increase of the cohort of older people can affect, among other aspects, the amount and age structure of the workforce [20], the inequality and risk of poverty [21], the electoral participation [22,23], the economic activity [24] or the fiscal sustainability of the welfare state [25]. In any case, there is a need for more research on the benefits and potential of an aging society [26].

Among the many relevant researches devoted to widen the capacity of neoclassical economics to understand increasingly complex socioeconomic processes, this paper provides a more comprehensive and balanced understanding of aging, one of the most challenging current phenomena. Specifically, it answers the next research question: Compared to the rest, are aging societies bound to a worse economic performance and a lower capacity for reforms?

In order to answer this question and based on the civil economy approach, this paper discusses and calls into question several "myths" and widespread ideas in the economic literature on demographic aging and, specifically, on three "myths" enunciated by the Joint Academy Initiative on Aging [15], which have a relevant economic impact: first, the supposed negative influence of population aging on economic growth; second, its impact on labor productivity and, finally, the almost irreversible impact of aging on the economic sustainability of the public pension schemes and public health systems, two pillars of the welfare state, a complex organization regarded as impossible to transform in order to respond to a new demographic structure change. In addition, it presents a question that, based on the existing literature, might be an open question in the field of population aging and its economic consequences: the possible relationship between this phenomenon and labor productivity in developed economies. 

Thus, based on the civil economy framework and beyond the neoclassical economic approaches, this paper contrasts the following hypothesis:

*Compared to the rest, aging societies can show the same economic performance and capacity for reforms.*

In turn, this statement presents two main sub-hypothesis.:

- Aging societies are as capable as other societies of carrying out long-term reforms in order to ensure the sustainability of the system.
- An increase of the average age of the population does not have to lead to a weaker economic growth and less productivity.

The capacity of a society to implement reforms, and to ensure economic growth and higher productivity levels are three of the main key socioeconomic factors for a sustainable development. Beyond the short-term, these three aspects (reforms, growth and productivity) are important for the environmental health and the quality of life, given that economic growth and productivity affect living standards [27], and the reform of public health and pensions systems are also a crucial field of research for health economics [28]. The analysis framework of the civil economy is a useful economic research framework that employs a humanistic approach and provides a comprehensive understanding of the complex and multifaceted phenomenon of population aging [29].

In order to conduct this research, a qualitative methodology with an inductive approach is used. This qualitative and inductive methodology is displayed throughout the whole article. In a first stage, the authors explore the existing analyses that link aging societies with economic performance and the ability to introduce long-term reforms. Although a part of this exploration is presented at the beginning of the introduction section, the complete exploration is introduced in Section 2. As a result of the contradictory data and evidence found in that exploration, in a second stage, the authors present a research question and a hypothesis in order to clarify the different views identified in the exploration. In a third stage, the authors aim to find new outcomes able to make a contribution in the field of study related to the research question. This task is performed in Section 3, analyzing the three “myths” identified in the literature that link aging societies with poor economic performance and poor capacity to implement long-term reforms. In a fourth stage, the authors reach to some general conclusions based on all prior data and analyses. This task is displayed in Section 4, Conclusions. 

At the same time, the authors use the framework of the civil economy in order to analyze all the evidence gathered in Section 2. In this way, in Section 3 the authors can provide answers to the 3 myths identified in the literature which are based not only in the market and the egotistic behavior of individuals but also in the capacity of cooperation between different institutions (public administrations, corporations, health care systems) and between persons. In Section 4, the possibility of combining cooperation and self-centered behavior in order to facilitate new insights in this field of study is highlighted.

The main contribution of this research lies in its effort to keep fostering a more balanced perspective on population aging and to call into question some of the economic implications of this phenomenon. It is also, as far as the authors are aware, an innovative and structured effort to analyze population aging from the prism of the civil economy paradigm [29]. 

The paper is structured as follows: after introduction, in Section 2, the research and reference literature is presented; then, in Section 3, certain ideas and “myths” about the economic effects of the aging phenomenon are discussed, a possible connection between aging and labor productivity is presented as an open question, and implications for theory, policy and public health are explored. In the final section, main ideas are exposed in relation to the subject under analysis, as well as possible areas of future research to continue putting population aging into perspective. 

## 2. Research and Reference Literature

In this Section 2, and following an inductive approach, the authors are going to explore evidence and data presented by a variety of researchers in the field of the research question of this work. As stated in the introduction section, this paper analyses population aging adopting a more balanced vision based on recent research. In this spirit, there are authors who defend a non-alarmist view of demographic analysis, as well as an understanding of the population aging phenomenon, adopting a revised and systemic perspective. Among them, the following can be highlighted: the early analysis about aging and macroeconomic change by Cutler, Poterba, Sheiner and Summers [30], the critical evaluation on demographic alarmism done by Vincent [31], the development of the "longevity dividend" concept [32,33], several papers on demographic decline and population aging [34,35], the ideas of Börsch-Supan [36] about aging and well-being, the work done on the "successful aging” concept [26,37], the analysis regarding the relativity of chronological age and images of aging [17] and, last but not least, the interesting report published by the Joint Academy Initiative on Aging [15], a clear and structured effort to refocus the debate on population aging in Germany and, by extension, throughout Europe and other countries. These references have been a relevant compass for the present research and its development.

The research mentioned above follows a non-alarmist or "revisionist" position in demographic analysis [14]. A trend produced because of the arrival of people with economic training to the demographic analysis during the second half of the 20th century, in a field that had previously been dominated by researchers from the fields of biology and natural sciences. This "revisionist" perspective presents three assumptions:

- Focus on the medium and long term;
- Evaluation of the multiple positive and negative effects;
- Consideration of the indirect effects of demographic changes.

These assumptions make the “revisionist” research agenda very demanding [11], since it takes into account a wide variety of forces and long time periods to carry out its analyses and contrasts. An approach that makes the "revisionist" school conclusions more moderate. This "revisionist" approach not only analyzes demographic increases, but it also tries to understand population aging and decline. In short, the gradual shift from an "alarmist" to a "revisionist" perspective in demographic analysis involves a lesser extreme debate, and it broadens the approach to describe, analyze and understand demographic phenomena. 

Regarding the civil economy framework, it is a paradigm that sinks its roots in the civil humanism and the Italian economic thinking of the eighteenth century [38,39] and, after enjoying some success, was replaced by the political economy framework [40]. 

In particular, the paradigm of the civil economy emerged in the 18th century thanks to the work of the Neapolitan philosopher Antonio Genovesi [38]. This approach advocates not only a relational and social economy but also a cooperative approach to the market. [41] The cornerstone of the Neapolitan school of civil economics is the belief that human beings develop reciprocity, an aspect rooted in human nature [42,43]. In fact, civil economy can be understood as a "political economy of virtue" [44] (p. 582).

The understanding of the human nature underlying the civil economy can be found on the personalist anthropology [45]. According to Bruni and Zamagni [46] human beings are not only individuals but persons. This anthropology recognizes a certain level of individualism and, at the same time, places the person out of the individualistic mode [47]. This point of view states that human beings are capable of behaving as individuals and/or persons. As individuals, human beings try to maximize their own interests. However, Personalism goes beyond this limited view and proposes that human beings are also persons, which are relational in nature and are able to cooperate in common projects (corporations, foundations, institutions) at the service of society [48,49]. In short, the personalist approach provides a holistic understanding of the human being, able to transcend his/her own individuality and cooperate with others, also in the economic sphere [46,50].

The civil economy perspective not only goes beyond the standard economic approach and its reductionisms but also widens the political economy approach, which defends a “two-hand” system composed by the invisible hand of the market and the visible hand of the institutions. The civil economy perspective evolves this view towards a “four-hand" system, and it also takes into account active citizenship and productive and sustainable organizations, in addition to market and institutions [51]. In this sense, the civil economy paradigm seeks to transcend the reductionist vision that the neoclassical paradigm has of individuals, corporations and value, to focus on generativity and the common good [52,53,54,55]. 

In the demographic aging field, it means going beyond the “political economy of aging” approach to evolve towards the “civil economy of aging” perspective. The former focuses on analyzing the social, political and economic processes of aging; the production and distribution of scarce resources and the role of the State and the market [2,56,57], while the latter takes into account a more holistic and less reductionist vision, by including active citizenship and sustainable productive organizations in the analysis [29].

As stated above, the civil economy is a useful tool for humanizing the economy [58], taking into account the philosophical roots of the limits of our socioeconomic system. Moreover, the humanization of the economy is relevant for a better understanding of population aging phenomenon. This is why the civil economy approach is proposed as a theoretical framework for analyzing the complex and multifaceted phenomenon of population ageing [29]. This effort is based on the fact that interest in the various theories that are used—or can be used—to understand the aging phenomenon has resurfaced over the last three decades [59,60].

In addition, the civil economy paradigm can be useful in overcoming the "level myopia" suffered by the theoretical frameworks of aging [61], i.e., the excessive focus on the micro level and on individuals, without taking into account the repercussions at the macro or aggregate level. In this line, this approach entails taking into account that demographic ageing not only has an impact at the institutional (e.g., public health, pension system) and market economy levels (e.g., economic growth) but also affects individuals (e.g., productivity) and enterprises (e.g., automation). By considering the “four hands”, the civil economy offers the possibility of connecting the micro and macro levels. This is a useful aspect for this research, since its main objective is to offer a more comprehensive and balanced understanding of the effects of aging on the economy.

## 3. Myths, Beliefs, Challenges and An Open Question

In Section 3, and continuing with the inductive approach, the authors aim to find new outcomes able to make a contribution in the field of study related to the research question, specifically regarding the three “myths” identified in the literature. As mentioned above, population aging causes a wide range of socio-economic effects. These effects are often analyzed through the lens of a somewhat “alarmist” or, at least, negative perspective. As Staudinger [17] observes, negative associations regarding old-age still prevail in research about old-age stereotypes. 

It seems that there are many "myths", wrong beliefs and a lack of understanding around the demographic aging phenomenon. Rowe [62] argues that American society believes in ideas regarding aging that are—partially or completely—false and defends that these "myths" must be analyzed and unraveled. Bösch-Supan [16] also states that population aging is full of beliefs that must be analyzed in a scientific manner, with the aim of "demystifying" them. Both authors expose a list of wrong beliefs, such as the idea that an increasing immigration can stabilize the population average age, aging is a temporary phenomenon or older societies have more intergenerational conflict. 

For its part, the Joint Academy Initiative on Aging [15] exposes and refutes part of these “myths” in its report "More Years, More Life". The Joint Academy Initiative on Aging is a group of scientists, scholars and practitioners from interdisciplinary fields [63] such as “history, computer science, medicine, neurosciences, economics, philosophy, political science, psychology, law, and sociology as well as regional studies and engineering” [15] (p. 9). One of the main contributions of their research is to provide a systemic approach to demographic change. 

The Joint Academy Initiative on Aging analysis are important not only to foster a productive aging in aging societies but also to help individuals have a worthwhile and healthy later life [64], both in Germany and abroad. The research started in 2006, its conclusions were compiled in 2009 and the main findings were translated into English in 2010. Since then, the initiative has helped drive population aging research [65,66].

This article focuses on developing and debating three of the fifteen "myths" enunciated by this initiative. These three specific “myths” are chosen because their analysis and discussion help to answer the research hypothesis and two main sub-hypotheses raised in the introduction section. Furthermore, these “myths” have a special impact from an economic perspective, namely:

- Myth 15: “Aging societies are incapable of reform”;
- Myth 7: “Economies with an aging population are doomed to zero growth”;
- Myth 4: “Older employees are less productive”.

The present section begins with an analysis on the influence of demographic aging on public pension and health systems, to show examples of reform in order to ensure their viability in the context of aging societies (considerations about myth 15). Subsequently, it analyzes the existing literature on population aging and economic growth, as well as recent research that draws interesting conclusions about this issue (considerations about myth 7). Then the section addresses an evolving issue in the scientific literature: the possible relationship between population aging and different levels of labor productivity (considerations about myth 4). Finally, implications for theory, policy and public health are explored. The following pages will discuss population ageing at a macro level, so they present the consequences of this phenomenon at a high level of aggregation, rather than its impact on individuals. In any case, it should not be forgotten that aging is a multifaceted phenomenon [67] and influences the micro and macro levels, the former being "nested" in the latter.

### 3.1. Are Aging Societies Incapable of Reform? The example of Public Pensions and Health Systems throughout Europe, Japan and China (Considerations about Myth 15)

The literature has extensively analyzed the possible effects that population aging can have on public pension systems [13,19,25,36,68,69], and there is consensus that there will be a significant impact [13,70].

The influence of population aging on public pension systems is twofold. Not only life expectancy increase makes the beneficiaries to live longer, and therefore, the resources that must be dedicated to them increase [71], but also in the medium term, the fertility rate decline causes fewer new entrants to the labor market, which precipitates a decline in revenues for public pension schemes. In any case, there are voices who believe that the impact of aging in the pension system can be mitigated—or even neutralized—[71,72,73] implementing the appropriate policies and reforms well in advance. These authors argue that misperceptions exist about the age of retirement [15], the chronological time and intrinsic time [74], the conception of the system of distribution in pensions or the relationship between longevity and senescence.

First, the retirement age—established in many European countries at the age of 65 or less [75]—is a social construct that must be modified and expanded in the light of significant increases of life expectancy experienced in recent decades. Furthermore, the period of senescence has also been significantly postponed [13,76]. 

Therefore, there are authors who defend a relationship between life expectancy increase and the number of years a person can expect to live in good health [68]. In this context, during the last lustrum, many EU-28 countries have undertaken a slow but inexorable path to reform their public pension schemes, as well as to rise the retirement age in the coming decades [77]. After all, the ultimate goal of public pension systems should not be to retire at the age of 65, but to adapt the system to ensure its sustainability and maintain a scheme that guarantees decent pensions. 

Moreover, China has also been carrying out reforms on its pension system during the past decade, due to its fast population aging process [78,79,80]. Japan can also be an interesting example to move towards an increasing participation of older population in the labor market. In the land of the rising sun, the participation of the oldest cohorts remains high, unlike what happens in Europe, a continent that shows a declining participation of older people in the labor market. According to Clark, Ogawa, Lee and Matsukura [81], this has been possible thanks to reforms introduced in the Japanese system, such as the elimination of barriers to hiring older workers and the implementation of changes in the benefits of the social security system.

Regarding public health systems, a review of the literature shows how they can be negatively affected by the population aging phenomenon [25,69,77,82,83]. This conviction is motivated by the believe that older people tend to be the ones who use the most public health systems and who spend more on healthcare [84]. They usually endure physical and/or mental pain during the last stages of their life and must be institutionalized or cared for by their relatives as they fall into dependency [69]. In any case, to call into question this perspective, it must be said that older people have proven to be better providers than recipients of aid up to the age of 80 [15], an idea rarely highlighted in the economic literature. An example of this is the fundamental contribution to their children and grandchildren, both in monetary terms and care, after the serious economic crisis suffered by Spain in 2008 [85].

In any case, it seems that the aging process could further increase public health expenditure of a large part of EU-28 countries [77]. This increase can be contained through a series of measures to rationalize and improve the efficiency of the system, such as the promotion of healthy living habits to reach the period of old age in the best possible health condition [82]. Another way to contain and manage the probable increase in public health spending is the evolution of the European health systems to better consider and manage chronic conditions, such as diabetes or hypertension, among others. In other words, demographic aging leads to an increment in health problems related to chronic diseases, a trend that could emerge as one of the most expensive challenges for health systems [86,87]. 

An interesting example of reform and adaptation is the one implemented since 2009 by the public health system of the Basque Country region, in northern Spain. The Basque Country is a little region that experiences a pronounced process of population aging [88]. During its 9th Legislature, the Basque Government implemented a system-wide transformation of the public health care system, which serves to a population of approximately 2.1 million citizens. The main goals were to guide the system towards the care of chronic diseases, to reduce its fragmentation and to ensure its sustainability [89]. This strategy has given good results, and it continues today [90]. Besides, the population aging process means fewer young individuals, so there may be savings in health spending that can be redirected. Governments can relocate these health cost savings of the youngest age to meet the growing health needs that older cohorts have.

The existing literature regarding the impact of the aging process on public pension schemes and public health systems suggests that both may suffer tensions due to this trend. One way to respond to these tensions is to start implementing measures aimed at updating these systems. Bloom, Canning and Fink [13] (p. 588) argue that policymakers in European countries should take advantage of the "window of opportunity" that opens between 2010 and 2030 to prepare institutions as well as the welfare state in the face of the profound demographic change brought about by the massive arrival at the retirement age of the entire “baby boom” generation. This concern to take advantage of the years prior to the inevitable significant increase of people over 64 years in the EU is also shared by the European Commission [91] (p. 32), which instead indicates that this "window of opportunity" would be more effective if the aforementioned changes are implemented between 2010 and 2020. Besides, Heller [92] (pp. 16, 21), analyzing the case of Japan as the oldest society on the planet, also concludes that the country must take advantage of its corresponding "window of opportunity" between 2015 and 2025–2030, in addition to pointing out that countries must update and prepare their social security systems as they begin to move towards an aged society.

From the perspective of the civil economy, the construction of the welfare state and, in particular, the deployment of public health and pension systems have contributed to improving the living conditions of the population and, ultimately, to a more humane civil and economic organization. The fast and global sustained increase in life expectancy at birth would be a result of this socio-economic improvement.

As exposed above, aging societies are already implementing reforms in this direction [77,78,79,80,90], such as updating retirement systems in European countries, China and Japan or implementing a strategy of chronic diseases in the public health system of the Basque Country region, in Spain. From the perspective of the civil economy, the "hands" of the public administration and active citizenship drive these reforms, since the sustainability of the pension and public health systems is a recurrent demand of organized civil society, and the measures to make this possible have been devised and implemented by public administrations. Furthermore, all these efforts aim to update and preserve systems that are essential to the common good, an objective that is in line with the ultimate goal of the analytical framework of the civil economy [38,44].

These facts, such as putting into perspective the influence of population aging on health and pensions systems, can throw a new light on aprioristic assumptions and show that aging societies are capable of reform.

### 3.2. Are Economies with an Aging Population Doomed to Zero Growth? Aging and Automatization, a Possible Game Changer (Considerations about Myth 7)

Regarding the influence of population aging on economic growth, most of the existing literature defends a negative relationship between these two variables [13,24,93,94]. Many authors support this idea because changes in the demographic structure affect consumption, savings, productivity and/or the aggregate supply of labor.

Adopting a life cycle perspective, Bloom, Canning and Fink [13] argue that demographic aging may be accompanied by lower consumption, a higher rate of savings, deterioration in productivity levels and/or a lower participation of the labor force. Processes that could negatively influence economic growth. This thesis is also defended by Fehr, Jokisch and Kotlikoff [95] who, analyzing through a model the possible future evolution (2005–2100) of five large world areas (USA, Euro Zone, Northeast Asia, China and India), predict that long-term projection of US, Euro zone and Japan GDP may be damaged by the evolution of their demographic structures towards older societies.

The connections between population aging and economic growth are complicated. Various authors [27,96,97,98,99] argue that there are multiple factors that influence growth both positively and negatively. On the one hand, among the factors holding back growth are, for example, periods of war, financial crises or demand shocks. On the other hand, advances such as breakthroughs in health, education system reforms, the evolution of the economy toward knowledge-intensive sectors, and/or improving the skills and abilities of the workforce spur economic activity. The humanist vision of the civil economy aligns with the latter aspects and the importance it attaches to the microeconomic level, where the training, progress and wellness of people play a fundamental part in achieving higher levels of social prosperity.

Anyway, some authors argue that the adoption of an "alarmist" position is not justified [13]—neither the announcement of an economic catastrophe in the medium term—despite the possible negative effect between aging and economic growth. On the contrary, they trust that world economy has enough resources to absorb significant changes in the global demographic structure. Serow [69] also adopts a position more aligned with the "revisionist" point of view when analyzing demographic changes, since he defends that current economic structure has the flexibility and mechanisms to respond successfully to the challenges posed by population aging. On their part, van der Gaag and De Beer [5] also point out that, although demographic aging may present challenges at the macroeconomic level, there may be specific solutions by country to overcome them.

Acemoglu and Restrepo [100] have also analyzed the effects of population aging on economic growth in a working paper published by the National Bureau of Economic Research. Their work throws new conclusions taking into account the existing literature on the subject. The authors begin their analysis by pointing out that the thesis of "secular stagnation"—exhibited at the time by Alvin Hansen [101]—defends that the economy can stagnate for a long period of time due to the fact that aging creates an excess of savings in relationship with investments, which in turn would damage economic growth. Acemoglu and Restrepo research analyzes 168 countries between 1990 and 2015 with the objective of checking if population aging affects economic growth.

The results of their analysis are counterintuitive given that, not only they do not find a negative relationship but also because they find—according to their calculations—a positive and significant relationship in some of the cases analyzed. The authors expose that an explanation of this possible and unexpected relationship is that a replacement of labor by technology began to occur in the developed economies in the 1990s, especially through the implementation of robotics and artificial intelligence in their productive processes.

**Figure 1:**
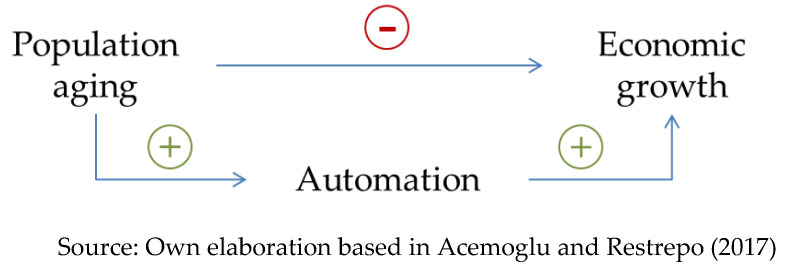
Diagram of relations between demographic aging and economic growth proposed by [100] Acemoglu and Restrepo (2017).

Therefore, although a higher average age in an economy could damage economic growth at first, it also seems to drive the substitution of labor factor for capital factor, which ultimately leads to automation and greater economic output (see Figure 1). In order to defend this thesis Acemoglu and Restrepo carry out the corresponding quantitative contrasts, finding a significant positive relationship between the aging process of the countries analyzed and the adoption of robotics.

Furthermore, according to the civil economy framework, the evolution of the economy towards greater automation of its productive processes can be understood as the correct functioning of the "hands" of the market and productive and sustainable enterprises, given that they generate the appropriate mechanisms to counteract a possible negative impact of ageing on economic activity. Companies with long-term vision would opt for investments in automation and new technologies to maintain productivity in the face of an increasingly scarce and aging workforce. These investment processes at a microeconomic level would not be possible without a market economy that channels savings towards investment and rewards those companies that embark on processes of productive improvement and innovation.

These findings and conclusions can put into perspective the claim that demographic aging harms growth and that the countries that experience it are "condemned" to zero growth.

### 3.3. Are Older Employees Less Productive? An open Question in the Economic Literature (Considerations about Myth 4)

An important aspect of Acemoglu and Restrepo idea is that countries facing a lower availability of labor due to aging processes can begin using capital factor with greater intensity, for example through processes of automation, to the detriment of an increasingly scarce labor factor. This greater capital input can have effects on output, which in turn leads to reflection on the possible impact of population aging on labor productivity.

"Myth 4" exposes that the individual productivity of workers does not necessarily decrease with age, since the productivity depends on the different abilities a job requires, and how they match with those of the people who carry it out [15]. When analyzing the available literature, there are analysis showing how no significant relationship is found between the age structure of the workforce and a decrease in productivity in the manufacturing, metallurgical and service sectors of the German economy [102]. Moreover, there are voices defending that the German population aging needs not automatically entail a threat to the overall productivity of the economy [15]. In addition, a research regarding age structure of the workforce and performance of Danish firms also rules out a negative relationship [103]. Finally, Börsch-Supan [16] (p. 10) argues that, although young workers are different from older ones, they are very alike in terms of productivity.

On the other hand, there are authors such as Feyrer [104,105] who argue that changes in the age structure of the labor force are strong and negatively associated with productivity and output, after analyzing 87 countries between 1965 and 1995. The author points out that one possible explanation may be the relationship between innovative activity and age. In any case, Feyrer admits the possibility that the age structure effect on productivity may decrease if the analysis is carried out at lower levels of aggregation, such as the regional, sectoral or urban level. In turn, three members of the European Department of the International Monetary Fund [106] published a working paper analyzing the effect of population aging on labor productivity in European countries between 1950 and 2014. They point out a negative and significant effect between these two variables. A recent research on Aiyar, Ebeke and Shao’s work, even if it also identifies this negative relationship for the period 1983–2014 in European countries, also presents some new questions about the evolution and possible disappearance of the effect of population aging on labor productivity during the 1995–2004 decade [99]. Anyhow, the literature investigating a possible relationship between population ageing and aggregate productivity is "surprisingly small" [107] (p. 3).

Taking into account the different existing perspectives, it is interesting to highlight that Lindh [108] exposes the difficulty of answering the question of whether population aging affects productivity, given that productivity measures themselves—as Total Factor Productivity (TFP)—are controversial within the economic field and hard to grasp [16]. Another relevant issue put forward by Lindh is that one must choose and explain carefully at what level productivity is measured, since it can be at an individual, productive plant, industry, national or global economy level. Moreover, Lindh warns that “a priori” conclusions in relation to the subject of study are not very useful, reason for which he rejects the assumption of a possible negative relationship between aggregate labor productivity and demographic aging. Regarding the concept of productivity itself, Staudinger and Bowen [109] argue that it should be used in a wider way, given that in the work context, people are not only economically productive but also intellectually, emotionally and/or motivationally productive.

Based on these references and the diversity of existing opinions, it seems relevant to continue reflecting about and researching on the possible influence of population aging on labor productivity at the individual, firm, industry and aggregate levels, since the case may arise where each level of analysis behaves differently. Better understanding of this possible relationship between population aging and productivity, which escapes the focus and scope of this paper, can be a relevant research area. Anyway, when analyzing the possible different behavior of each level, it is important to remember the need not to fall into the aforementioned "level myopia" or an excessive focus on the micro level without taking into account the repercussions at the aggregate level.

In this sense, it would be interesting to use the civil economy framework to not to fall into the “level myopia” and to broaden the concept of productivity to include valuable activities that are not usually monetized and that are carried out by active citizenship (e.g., community work or care of dependents) [110]. This approach fits in with the civil economy’s efforts to overcome the reductionist approach that the neoclassical paradigm gives to the concept of value, which focuses on profit maximization by corporations.

For all the above, it is considered that the statement "older employees are less productive" (myth 4), could be an open question and an interesting field of research to continue advancing in the understanding of the population aging consequences. In the following section, some policy implications around the three “myths” exposed are discussed.

### 3.4. Implications for Theory, Policy and Public Health

Regarding myth 15 “aging societies are incapable of reform”, Section 3.1 has presented many countries undertaking reforms. According to the report of the Joint Academy Initiative on Aging, political preferences, worries about the future sustainability of each society, health systems and pension funds are topics where is not possible to find a clear division between older people and the younger cohorts [15]. Empirical evidence seems to support the idea that active engagement of older people in society tends to equalize political choices in relation to the entire society [15], making possible important reforms, such as the ones related to health systems in aged societies [70,77,78]. In order to make possible this engagement, measures of public health tending to postpone the senescence period could be useful [15,57]. Those measures can be implemented since the early schooling period, entering in the curricula basic aspects about nutrition, lifestyle and positive advice to avoid chronic diseases. Theoretical frameworks linking the introduction of public health advices in early education with the achievement of longer lives with better quality could be developed and their results applied in policy recommendations.

In relation to myth 7 “economies with aging population are doomed to zero growth”, the research presents macroeconomic evidence that acknowledges this linkage [13,24,93,94], whereas other authors [5,69] undermine any automatic relationship between growth and aging population, and others moderate this relationship through automation [100]. From a macroeconomic perspective, if the percentage of inhabitants that is active declines in any given society, and at the same time, workforce productivity declines, the effect on economic growth will be negative [13]. However, public policy measures linked with public health provisions can be implemented in order to avoid the negative macroeconomic impact. 

In the first place, through effective public health interventions since early education, employees might be ready to invest some of their gained life years to continue being active as part of the workforce. This intention could be accompanied with legal reforms raising the legal retirement age and a legal framework making attractive for older members of the workforce to join mixed situations where they can make compatible part-time job involvement with receiving pension benefits [15]. In this way, aging societies could avoid the situation of losing (or even increasing) an important part of their workforce in a short period, with positive macroeconomic effects. 

In fact, in societies where public health measures are successful the “compressed morbidity” phenomenon has emerged, according to which preventive public health measures are able to postpone the period of senescence, procuring more years of active engagement in society to older people (through being part of the active workforce or voluntary work, for example) and reducing the time span between serious deterioration of health and death [15]. These findings are aligned with the revisionist point of view about myth 7 [5,69]. At the same time, the achievement of this compressed morbidity will make possible for older people to have higher levels of income (through longer professional careers) and to reduce the cost of health treatments for society and themselves.

Myth 4 states that older employees are less productive. Some authors agree with this affirmation [104,106], while others ask for a broader framework of analysis and do not agree that there is an automatic and negative relationship between productivity and age [15,16]. In this regard, there are some initiatives that can be taken at the level of the corporation, at the social level and at the individual level that may attenuate or even reverse the negative relationship stated in myth 4. At the level of individual institutions (corporations, public administration and others), the inclusion of life-long learning schemes throughout the entire working-life of the employee can have a constant positive effect on personal productivity, regardless of age [15]. This possibility could be supported by specific legislation drafted under the agreement of employers, trade unions and individual employees. In addition, corporations and other employers could plan lateral mobility in order to accommodate employees in tasks where they can be more productive in accordance to their age, a task that could be handled jointly by human resources departments and trade unions [15]. At the same time, corporations and employers should develop specific policies to facilitate a positive mental and physical situation for employees while they develop their professional tasks, in order to facilitate long-term productivity of employees when they reach an old age. Again, the collaboration between employers, trade unions, legislation and employees is necessary [111]. This broader framework dismisses a direct and negative relationship between productivity and age. However, more theoretical and empirical research is needed in this field, as suggested by recent studies [107].

## 4. Conclusions, Limitations and Future Lines of Research

In this Section 4, and as the final part of the inductive approach, the authors present general conclusions based on all prior data and evidence analyzed in the previous sections. 

The present paper analyzes and puts into perspective the population aging phenomenon, as well as the fact that the scientific economic literature approaches it in a narrow way and as a threat, despite being one of the greatest human achievements. The economic research addresses the socioeconomic effects of the older population’s growth with certain degree of "alarmism", and more knowledge about the benefits and potential of an aging society is needed. Therefore, the main objective of this paper is to throw light to some economic "myths" associated with population aging, and to keep promoting a more balanced view of this phenomenon. 

The analysis uses the civil economy framework, which goes beyond the neoclassical economic framework and the "political economy of aging", and takes into account not only a "two-hand" system (the invisible hand of the market and the visible hand of institutions) but a "four-hand" system (the previous two "hands" plus an active citizenship and sustainable productive organizations). As far as the authors are aware, it is an innovative and structured effort to analyze population aging from the prism of the civil economy framework [29]. 

This framework provides an interesting tool in order to analyze, with an inductive approach, the evidence gathered in the exploration (Section 2), to discuss "myths" and public health issues (Section 3) and to reach conclusions (Section 4). The strength of the civil economy lies in its anthropological assumptions around the human being. As explained in the paper, the civil economy framework allows to analyze and to provide recommendations based not only on markets and the optimization of personal choices but also on the possibility of cooperation among institutions and persons. This view is consistent with a revisionist and not alarmist position regarding aging societies and is not frequent in the economic literature studying the aging field. 

Furthermore, the present research keeps fostering a more balanced perspective on aging, since it is a field of research where significant efforts have to be made to offer a more comprehensive approach [62]. It also calls into question widespread ideas about the effects of aging on society and the economy, an approach that mainstream literature does not commonly address. Demystifying aging with scientific evidence is an important task within the economics of aging [16]. All these features distinguish the present research in comparison to others in this specific field of study.

Taking into account the academic literature explored in Section 2 and the discussions provided in Section 3, the main hypothesis of this paper ("Compared to the rest, aging societies can show the same economic performance and capacity for reforms") can be accepted.

Regarding the economic "myths" and widespread ideas surrounding population aging, this text has focused on three generalized economic beliefs, which are stated as sub-hypotheses 1 and 2 in the introduction section. First, in the idea that aging societies are incapable of undertaking structural reforms (sub-hypothesis 1), secondly, that they are condemned to low rates of economic growth and, finally, that this phenomenon damages their labor productivity (sub-hypothesis 2). The paper has taken as a reference the list of "myths" enunciated in the report "More Years, More Life", published in 2010 by the Joint Academy Initiative on Aging, as well as analysis done by other authors.

Although there is evidence that population aging has economic and social consequences, the text shows—based on the existing literature, recent research findings and specific examples—that many of the existing pre-conceived ideas about aging are not entirely accurate. Specifically, it exposes how there exists a misconception about the age of retirement, and how a gradual increase, as well as other reforms, could guarantee the sustainability of public pensions. In fact, most of EU-28 members and other countries as Japan or China have already embarked in this process. In relation to public health systems, the text shows how many researchers and analysts predict a negative and irreversible impact on them due to the increase of older cohorts. Anyhow, it is rarely noted that people under 80 years tend to be more providers than recipients of aid. An example of this behavior is the great contribution of older people to their families, both in monetary terms and care, during and in the aftermath of the serious economic crisis Spain suffered in 2008. In addition, there are also examples of smart transformation of public health systems to adapt them to the challenge of an aging population, such as the one the Basque Country region in Spain [71] is leading. The regional government is transforming the system to guide it towards the prevention and care of chronic diseases. These examples challenge the myth that aging societies are incapable of carrying out long-term reforms in order to ensure the sustainability of the system. This means that, according to the academic literature presented in Section 2, and the discussion in Section 3, it is possible to accept sub-hypothesis 1.

Besides, despite the fact that mainstream scientific literature defends that an increase of the average age of the population can damage economic growth and productivity, the publication of a research by Acemoglu and Restrepo seems to put into question this widespread belief. Their hypothesis is based on the idea that, although at first population aging can negatively affect economic growth, it also drives the automation of the productive enterprises of the economy in question, causing a greater economic output generated in the economy as a whole. This recent line of research could call into question the “myth” that aging condemns economies to poor economic growth. 

In addition, the research carried out by the Joint Academy Initiative on Aging calls for life-long learning through all the working life, lateral movements of employees inside corporation according to their age, public health initiatives in order to protect the health of employees and a careful development of human resources policies in order to avoid burn-out situations for employees and protect their mental health. All those initiatives could enhance the productivity of employees when they reach an older age. This means that it is possible to accept sub-hypothesis 2.

Regarding the theoretical and policy implications of this research, policy makers must take advantage of the "window of opportunity" opened between 2010 and 2030 to prepare institutions and the welfare state to face the massive arrival at retirement age of the baby boom generation originated after World War II [13,91,92]. From an economic point of view, advanced economies should foster the evolution of their economies towards knowledge-intensive sectors in order to grasp the full potential of their aging workforce. This includes life-long learning through all the working period of employees inside the corporation [99]. At the same time, and following the advice of the Joint Academy Initiative on Aging [15], measures of public health should be promoted to protect the physical and mental health of employees in the long term, both inside and outside the corporation. Finally, with regard to progress in research on ageing, an effort should be made to achieve metrics that reflect the physical and cognitive age of individuals, beyond their chronological age [17] (p. 192).

This paper has sought to address certain "myths" that economic literature seems to produce and reproduce in the field of population aging, but its focus has been limited to the areas exposed. It would be interesting to address a more holistic and interconnected vision—beyond the reforms of the welfare state, economic growth and productivity—on the variety of economic wrong beliefs that this phenomenon presents. The possible connection between population aging and labor productivity at the aggregate level could also be a relevant area for further research. Finally, further analysis on the benefits and potential of an aging society can be an interesting future line of research. One of the main limitations of this study is the lack of econometric contrasts of the hypotheses that have been formulated. This econometric contrast could be made taking into account not only the economic characteristics of given regions or countries, but also their institutional arrangements.

Considering all previous discussions, the humble answer to the question of this paper’s title is that economic research should not fear population aging. Accordingly, further research on the positive aspects of aging can keep putting into question the "myths" that surround this phenomenon, as well as keep studying its multifaceted effects from a non-aprioristic approach. The understanding of these questions is of vital importance in order to make population aging not only a "pleasant macroeconomic experience" [36] (p. 406), but also a kind social transformation in which the "four hands” of the civil economy intervene to foster the common good and a "livable aging society" [112] (p. 524).
